# Growth Inhibition of Sulfate-Reducing Bacteria in Produced Water from the Petroleum Industry Using Essential Oils

**DOI:** 10.3390/molecules22040648

**Published:** 2017-04-19

**Authors:** Pamella Macedo de Souza, Fátima Regina de Vasconcelos Goulart, Joana Montezano Marques, Humberto Ribeiro Bizzo, Arie Fitzgerald Blank, Claudia Groposo, Maíra Paula de Sousa, Vanessa Vólaro, Celuta Sales Alviano, Daniela Sales Alviano Moreno, Lucy Seldin

**Affiliations:** 1 Instituto de Microbiologia Paulo de Góes, Universidade Federal do Rio de Janeiro, Rio de Janeiro 21941-590, Brazil; pamellaethuany@gmail.com (P.M.d.S.); fatimagoulart@micro.ufrj.br (F.R.d.V.G.); jomonteza@yahoo.com.br (J.M.M.); alviano@micro.ufrj.br (C.S.A.); danialviano@micro.ufrj.br (D.S.A.M.); 2 Instituto de Ciências Biológicas, Universidade Federal do Pará, Pará 66075-900, Brazil; 3 Embrapa, Agroindústria de Alimentos, Rio de Janeiro 23020-470 Brazil; humberto.bizzo@embrapa.br; 4 Departamento de Engenharia Agronômica, Universidade Federal de Sergipe, Sergipe 49.100-000, Brazil; afblank@ufs.br; 5 CENPES/Petrobras, Rio de Janeiro 21941-915, Brazil; groposo@petrobras.com.br (C.G.); mpsousa@petrobras.com.br (M.P.d.S.); 6 Grupo Falcão Bauer, São Paulo 05036-070, Brazil; vvolaro.falcao_bauer@petrobras.com.br

**Keywords:** essential oils, antimicrobial activity, sulfate-reducing bacteria (SRB), production water, oil industry

## Abstract

Strategies for the control of sulfate-reducing bacteria (SRB) in the oil industry involve the use of high concentrations of biocides, but these may induce bacterial resistance and/or be harmful to public health and the environment. Essential oils (EO) produced by plants inhibit the growth of different microorganisms and are a possible alternative for controlling SRB. We aimed to characterize the bacterial community of produced water obtained from a Brazilian petroleum facility using molecular methods, as well as to evaluate the antimicrobial activity of EO from different plants and their major components against *Desulfovibrio alaskensis* NCIMB 13491 and against SRB growth directly in the produced water. Denaturing gradient gel electrophoresis revealed the presence of the genera *Pelobacter* and *Marinobacterium*, *Geotoga petraea*, and the SRB *Desulfoplanes formicivorans* in our produced water samples. Sequencing of *dsrA* insert-containing clones confirmed the presence of sequences related to *D. formicivorans*. EO obtained from *Citrus aurantifolia*, *Lippia alba* LA44 and *Cymbopogon citratus*, as well as citral, linalool, eugenol and geraniol, greatly inhibited (minimum inhibitory concentration (MIC) = 78 µg/mL) the growth of *D. alaskensis* in a liquid medium. The same MIC was obtained directly in the produced water with EO from *L. alba* LA44 (containing 82% citral) and with pure citral. These findings may help to control detrimental bacteria in the oil industry.

## 1. Introduction

Oil exploration and production generates a large amount of produced water (defined as the water that exists in subsurface formations and is brought to the surface during oil and gas production) [1]. Typically, a new field yields about 5 to 15% produced water of the total volume of petroleum. However, as the field matures, the produced water volume can reach up to 90% of production because additional water is usually injected into the reservoir to sustain the pressure necessary to maintain or increase oil recovery levels [2].

At the surface, produced water is separated from hydrocarbons, but small suspended oil particles and dissolved organic and inorganic compounds (mainly chloride and sulfide ions) still remain, making the discharge of produced water an environmental problem [3,4]. Different strategies have been developed to reuse and recycle produced water, including re-injecting it back into reservoirs to increase oil production and many other industrial uses [1,5,6,7].

Water-injection systems are frequently contaminated by bacteria that can cause severe plugging of surface and downhole equipment and injection-well formations, and also generate H_2_S that can indirectly cause pitting corrosion [8,9]. Biogenic sulfide production (biogenic souring) results from sulfate-reducing bacteria (SRB, [10]) or other sulfidogenic bacterial activity. In addition to the corrosion of metal surfaces, H_2_S is both toxic and explosive [11,12]. To counter these effects, bacterial growth in water-injection systems is controlled mainly by chemical biocides, such as chloride, glutaraldehyde and quaternary ammonium salts [13,14]. Several other water-based fluids used in oil and natural gas drilling and production operations benefit from the use of biocides, as well as the water holding tanks. However, biocides may fail because of difficulties in penetrating bacterial biofilms and also due to bacterial biocide-resistance [15]. Moreover, residual concentrations, toxicity and persistence of biocides in industrial effluents are known to be detrimental to public health and the environment [14]. Hence, alternative biocides against harmful bacteria, with a particular focus on SRB control, are of great interest to the petroleum industry [14,16,17].

Several essential oils (EO)—complex mixtures of volatile, lipophilic and odiferous substances arising from the secondary metabolism of plants—have been used as therapeutic agents since ancient times, and some of them have been scientifically proven to possess medicinal properties [18,19,20] and antimicrobial activities [21]. They are mainly composed of monoterpenes and sesquiterpenes, and their oxygenated derivatives (alcohols, aldehydes, esters, ketones, phenols and oxides). Korenblum et al. [22] previously demonstrated that lemongrass (*Cymbopogon citratus* (DC.) Stapf) essential oil (LEO) or citral (the principal compound of LEO) is able to control planktonic growth of *Desulfovibrio alaskensis* strain NCIMB 13491 and/or prevent its biofilm formation and sulfide-induced corrosion of metal surfaces. However, though a wide assortment of EO have already been described in the literature, their antimicrobial activity against SRB growth is understudied.

In this study, we first characterized (using molecular methods) the bacterial community present in produced water samples from a Brazilian petroleum industrial facility. We then tested different essential oils and some of their major components against *D. alaskensis* (to assess control of SRB growth) and also directly, to elucidate their antimicrobial properties on our produced water samples.

## 2. Results

### 2.1. Bacterial Community in the Produced Water Samples

**Figure 1:**
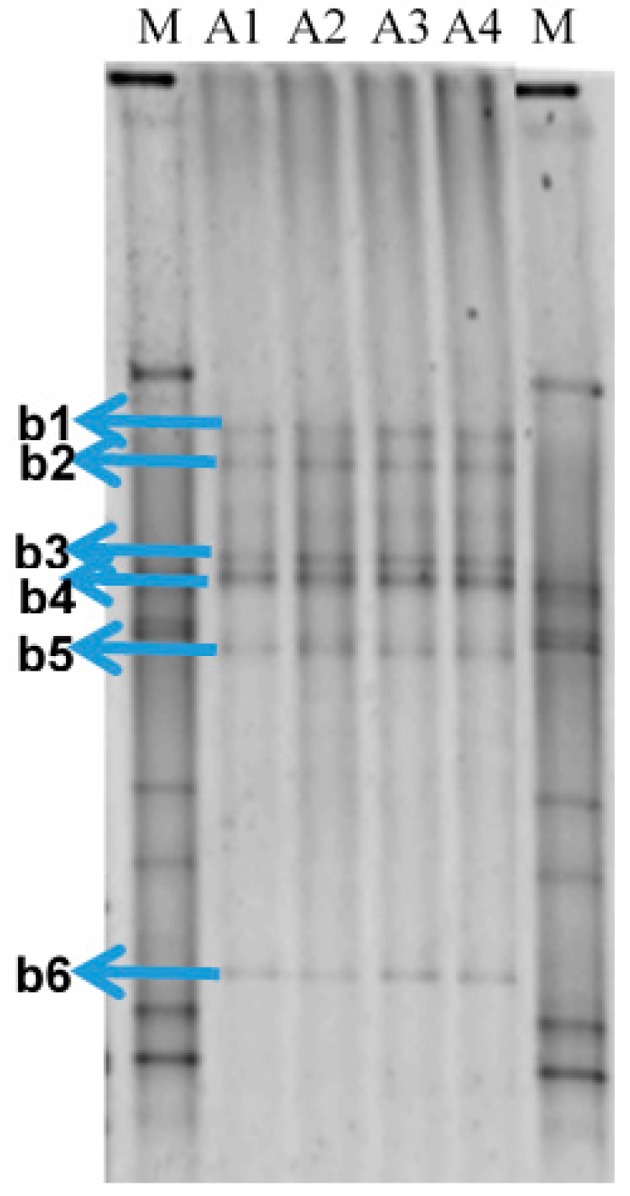
Denaturing gradient gel electrophoresis (DGGE) fingerprints to compare the bacterial communities of the four produced water samples received from Petrobras Ilha Grande Bay Oil Terminal (TEBIG). The letters **A** and **b** followed by a number correspond to the water samples and to the band retrieved from the gel that was subsequently reamplified and sequenced, respectively. The letter M indicates the bacterial standard marker.

DNA from the four samples of produced water received from Petrobras Ilha Grande Bay Oil Terminal (TEBIG) was analyzed by PCR followed by denaturing gradient gel electrophoresis (PCR-DGGE) of 16S rRNA-encoding gene fragments. Fingerprint analysis showed that the bacterial community structure was very similar between replicates (Figure 1). Unweighted pair group method with arithmetic mean (UPGMA) cluster analysis revealed that the profiles were clustered with a similarity >92% (Figure S1). Six dominant and common bands for the four profiles were extracted from the gel, cloned, sequenced and further identified (see Table 1). Nucleotide sequences have been deposited in GenBank under accession numbers: KY859414–KY859419. Two bands corresponded to the genus *Pelobacter* (belonging to the Deltaproteobacteria class, Desulfuromonadales order), one to *Geotoga* (Thermotogae class, Petrotogales order), and another two bands to the genus *Marinobacterium* (Gammaproteobacteria class, Alteromonadales order). Only one band corresponded to an SRB, which was identified as *Desulfoplanes formicivorans* (Deltaproteobacteria class, Desulfovibrionales order).

To further establish the diversity of the SRB population in the produced water samples, a portion of the *dsrA* gene coding for dissimilatory sulfite reductase was used to construct a clone library. All of the resulting sequences from the produced water sample (from 20 clones) were highly similar and associated with *D. formicivorans*, indicating that this sulfate-reducing bacterial species predominates in the produced water studied here. A representative *dsrA* nucleotide sequence has been deposited in GenBank under the accession number KY867755. Total SRB enumeration in the produced water sample was equivalent to 1.5 × 10^5^ MPN/mL.

### 2.2. Essential Oils and Major Components Against SRB

Table 2 shows the different plants from which EO were distilled and their major components. All of these EO were tested against SRB strain *D. alaskensis* NCIMB 13491 and also against produced water samples. Minimum inhibitory concentrations (MICs) determined using the crude EO against *D. alaskensis* varied from 78 to 2500 µg/mL (Table 2). The best results (MIC = 78 or 156 µg/mL) were observed for the EO extracted from “mirim” lime, “siciliano” lemon, lemon balm (*Lippia alba* LA10, LA22, LA44) and lemongrass (*Cymbopogon citratus*).

We also tested commercially-available major components of EO and found the lowest MIC values were observed for citral, linalool, geraniol and eugenol (Table 3). We selected those EO and their components exhibiting low MIC values to assess inhibition of SRB growth in produced water samples. We detected an increase in the MIC values (over our tests against *D. alaskensis*) for all EO or major components tested, except for *L. alba* LA22, LA44 and citral (Table 2 and Table 3).

## 3. Discussion

Various plant EO have been extensively studied because of their antimicrobial activity against a range of medically-relevant bacteria [21]. Furthermore, other biological activities have been attributed to different EO, such as anti-inflammatory, anti-diabetic, anti-mutagenic, insecticidal, molluscicidal and protozoicidal effects, among others [27]. Kerekes et al. [28] proposed the use of EO as natural preservatives and sanitizers in the food industry, and Borrego et al. [29] suggested their use in minimizing biodeterioration of documents. However, their potential for applications against detrimental bacteria in the petroleum industry is much less studied. Korenblum et al. [22] only evaluated the anti-corrosive effect and antimicrobial activity of one EO (lemongrass) against the growth of *D. alaskensis*. To our knowledge, the effects of other EO against SRB and their possible usage directly in produced water generated by the petroleum industry have never before been tested.

Our results demonstrate the potential to use different EO obtained from “mirim” lime, “siciliano” lemon, as well as different varieties of lemon balm and lemongrass, in the petroleum industry to control bacterial growth in produced water. The low MIC values (78–156 µg/mL) suggest that only a small amount of each EO is necessary to inhibit the growth of SRB, at least for the SRB model strain *D. alaskensis* NCIMB 13491 that was previously isolated from a soured oil reservoir [30]. MIC values for lemongrass essential oil (LEO) and citral of 170 µg/mL for the inhibition of SRB growth have been previously reported [22], and a comparable MIC value for LEO against other bacteria was presented by Adukwu et al. [31].

When we focused our MIC assays on the produced water samples from TEBIG, in general we found that higher concentrations of EO were necessary to inhibit SRB growth in produced water compared to *D. alaskensis* cultures, with the MIC value at least doubling for the majority of EO tested. This effect may be explained by the bacterial community present in the produced water samples, which may be interfering with the EO activity. Our molecular analyses revealed the presence in produced water samples of a mixed culture of four predominant genera, including the SRB *D. formicivorans*. *Geotoga* spp. are fermentative bacteria capable of reducing elemental sulfur to hydrogen sulfide [32], and *Pelobacter* spp. are commonly found in marine sediments [33] and oil reservoirs [34,35]. The presence of these two genera has already been demonstrated in anaerobic highly corrosive biofilms recovered from the inside of a steel pipe of an offshore oil production facility [36]. Sequences related to *Marinobacterium* have been found in the Xinjiang Luliang water-flooding petroleum reservoir [37]. *D. formicivorans* was recently isolated from the sediment of meromictic Lake Harutori in Japan and described as a novel species of a new genus belonging to the family Desulfomicrobiaceae [38]. Therefore, our results broaden the SRB targets that can be subjected to EO growth inhibition.

We also considered the major components of the different EO using commercially-available products, and our findings indicate that the presence of high concentrations of linalool, citral and a mixture of limonene and citral seemed to be responsible for the effectiveness of the various EO. The results we obtained for citral corroborate those obtained by Korenblum et al. [22]. Citral, linalool, geraniol and eugenol all presented low MIC values of 78 µg/mL when tested against *D. alaskensis*. As geraniol is a linalool isomer, the same response of *D. alaskensis* to both components was expected. Moreover, as eugenol (which is used as a major ingredient in a variety of dental materials) presents antibacterial activity mainly against anaerobic bacteria [39], SRB were also likely to be inhibited by it. However, only citral demonstrated equivalent effectiveness (i.e., low MIC) against bacterial growth in both pure SRB cultures and production water samples.

Our findings suggest that use of different EO and their major constituents may be an option for replacing or at least decreasing the application of synthetic biocides to control bacterial growth in the petroleum industry. We propose that the antimicrobial activities we observed directly in produced water samples will be the basis for further investigations of the use of EO as an option for controlling SRB growth in petroleum industry facilities.

## 4. Materials and Methods

### 4.1. Produced Water

Four receptacles containing 5 L of produced water were brought from TEBIG (coordinates: 23°3′23″ S, 44°14′6″ W), off the coast of Rio de Janeiro, Brazil. The water samples were maintained at 4 °C until use. The physicochemical properties of the produced water samples were: pH of 7.6, salinity of 23.2 g/L, Chloride 15.1 g/L, Calcium 0.51 g/L, Iron 1.2 mg/L, Potassium 0.16 g/L, Strontium 5.4 mg/L, Sulfide < 0.3 mg/L, Magnesium 0.45 g/L, Sodium 9.0 g/L, Barium 8.4 mg/L.

### 4.2. Sulfate-Reducing Bacteria

The sulfate-reducing bacteria (SRB) *Desulfovibrio alaskensis* NCIMB 13491 [30] was grown at 30 °C for 3–7 days in either sealed serum bottles or BD Vacutainer serum tubes containing, respectively, 10 mL and 1 mL of Postgate C or Postgate E media [40]. The bottles were purged with a N_2_ flux to achieve anaerobic conditions.

### 4.3. EO and Major Components

Fruits of different species of *Citrus* were collected in local markets in Rio de Janeiro, Brazil. Fruit peels (150 g) were removed and homogenized in distilled water using a laboratory blender. The resulting mixture was immediately subjected to hydrodistillation using a glass-type *Clevenger* apparatus as described in Simas et al. [23]. Lemongrass (*Cymbopogon citratus* (DC.) Stapf, Poaceae) and lemon balm (*Lippia alba* (Mill) N. E. Brown, Verbenaceae) leaves were collected from the Research Farm of the Federal University of Sergipe, Brazil. One sample of *L. sidoides* (LSID104) was collected at Poço Redondo, Sergipe, Brazil, and the remaining *L. sidoides* samples were harvested from the Research Farm of the Federal University of Sergipe, Brazil. Leaves from *Croton cajucara* Benth (Euphorbiaceae) were obtained from the Germoplasm collection of “EMBRAPA Amazônia Ocidental,” Amazonas, Brazil. EO of lemongrass, white and red “sacaca,” lemon balm and pepper-rosmarin were obtained from the fresh leaves by hydrodistillation [22,24,25,26].

All pure compounds of the major components of the EO used in bioassays were obtained from Sigma-Aldrich (São Paulo, Brazil).

### 4.4. DNA Extraction

A 50 mL sample from each receptacle containing produced water was filtered through a Millipore membrane (0.45 µm), before the total DNA was extracted using a FastDNA^®^ Spin Kit for Soil (MP Biomedicals, Santa Ana, CA, USA) and then stored at 4 °C prior to PCR amplification.

### 4.5. PCR Amplification of Bacterial rrs and dsrA Genes

PCR amplification of 16S rRNA-encoding genes was performed using the bacterial primer set U968f and L1401r [41] in a 25 μL-mixture containing about 10 ng of DNA, 0.8 µM of each primer, 0.5 µM of each dNTP, 2.5 mM MgCl_2_, 5 U *Taq* DNA polymerase (GOTaq^®^Flex–Promega, Madison, WI, USA), and 5 µL of the 5X PCR buffer supplied by the manufacturer. A GC-clamp was added to the forward primer [42]. The amplification conditions were as follows: 1× (3 min at 94 °C), 35× (1 min at 94 °C, 1.5 min at 55 °C, and 1 min at 72 °C), and an extension for 10 min at 72 °C. 

A portion of the *dsrA* gene coding for dissimilatory sulfite reductase was amplified using the primers DSR-1F [43] and DSR-R [44]. The 25 µL-reaction mixture contained about 10 ng of DNA, 100 nM of each primer, 0.2 mM of each dNTP, 1.25 U *Taq* DNA polymerase, 5X PCR buffer (Promega), and sterile Milli-Q water. The amplification conditions were as follows: 1× (15 s at 94 °C), 30× (15 s at 94 °C, 20 s at 54 °C, and 54 s at 72 °C), and an extension for 1 min at 72 °C. Positive (*D. alaskensis* strain NCIMB 13491) and negative controls (without DNA) were run in all PCR amplifications. 

The products were analyzed by electrophoresis in 1.4% agarose gels, followed by ethidium bromide staining (1.2 mg/L ethidium bromide in 1X TAE buffer-20 mM Tris-acetate, pH 7.4, 10 mM acetate, 0.5 mM disodium EDTA). 

### 4.6. Denaturing Gradient Gel Electrophoresis (DGGE) and Statistical Analyses

DGGE analysis was carried out as described previously [42] using an Ingeny PhorU2 apparatus (Ingeny International BV, Goes, The Netherlands). PCR products were loaded onto 8% (*w*/*v*) polyacrylamide gels in 1X TAE buffer. Polyacrylamide gels contained a denaturing gradient of urea and formamide varying from 46.5% to 60%. The gels were run for 17 h at 140 V and 65 °C. After this period, they were soak-stained for 1 h in SYBR Green I nucleic acid gel stain solution (10,000× *g* concentrated; Life Technologies, Eugene, OR, USA) and immediately photographed under UV light. Dendrograms were constructed based on the presence and absence of bands with the unweighted pair group method with mathematical averages (UPGMA) and the Pearson similarity coefficient using GelCompare II software (Applied Maths, Sint-Martens-Latem, Belgium).

### 4.7. Sequencing of DGGE Bands and dsrA Clone Libraries

Some bands were retrieved from the gels (marked in Figure 1) and 16S rRNA-encoding genes were reamplified as described above. These PCR products and those of *dsrA* PCR amplification were purified using Wizard SV Gel and PCR Clean-up System (Promega), and ligated to the pTZ57R/T plasmid vector using InsTAclone PCR Cloning Kit (Fermentas, Hanover, MD, USA), following the supplier’s instructions. The ligation products were transformed into competent *Escherichia coli* DH5-α cells. Sequencing of the inserts was performed by Macrogen (Seoul, Korea) using the primers U968F (16S rRNA) or DSR-1F (*dsrA*). 

The partial *rrs* gene sequences were identified using the BLAST-N facility (www.ncbi.nlm.nih.gov/blast) of the National Center for Biotechnology Information (NCBI) with the GenBank non-redundant database.

### 4.8. Enumeration of Sulfate-Reducing Bacteria

SRB enumeration in pure cultures and in the produced water samples was performed using the most probable number (MPN) method, as described by Postgate [40] and the MPN Reference Table [45]. The four water samples were combined to perform the MPN analysis.

### 4.9. Determination of Minimum Inhibitory Concentration (MIC) of EO and Major Components

Macrodilution susceptibility tests were performed in Postgate E broth (1990 µL) in BD Vacutainer tubes, with the different EO or their major components (10 µL) being added to the first dilution tube. The tube contents were serially diluted twice to a lowest concentration of about 19.5 µg/mL of EO or their components to determine the minimum inhibitory concentrations. *D*. *alaskensis* was grown for 3–7 days at 32 °C in Postgate C medium to yield a final SRB inoculum of 10^5^ cells/mL. A volume of 100 µL of the SRB culture (or the production water) was introduced into the various Vacutainer tubes. They were incubated for 7 days at 32 °C. SRB growth was detected by observing accumulation of the blackish color of the medium caused by iron sulfide precipitation in Postgate E medium. The minimum inhibitory concentration (MIC) was determined as the least amount of EO or major component added that did not result in a blackish color in the medium.

## Figures and Tables

**Table 1:** Identification (using the Basic Local Alignment Search Tool—BLAST-N) of partial *rrs* gene sequences of the different bands extracted from the DGGE gel.

Bands	Closest Database Match to A Cultivable Bacterium (Accession Number)	Identity (%)
B1	*Pelobacter* sp. (CP000142.2)	98
B2	*Geotoga petraea* (L10658.1)	100
B3	*Desulfoplanes formicivorans* (LC017841.1)	99
B4	*Pelobacter carbinilicus* (U23141.1)	98
B5	*Marinobacterium* sp. (KU052621.1)	99
B6	*Marinobacterium* sp. (HG315015.1)	99

**Table 2:** Determination of the minimum inhibitory concentration (MIC) of essential oils (EO) against *Desulfovibrio alaskensis* NCIMB 13491 and against sulfate-reducing bacteria (SRB) growth in produced water samples.

Plant	Popular Name	EO Distilled from (Plant Part)	EO Major Components	Reference	MIC of EO Against *D. alaskensis* (µg/mL)	MIC of EO in Produced Water (µg/mL)
*Citrus aurantifolia*	“Mirim” lime	fruit peels	31% R-Limonene, 8.5% β-pinene, 16% citral	[23]	78	156
*C. latifolia*	“Tahiti” lime	fruit peels	35% R-Limonene, 5% β-pinene, 5% citral	[23]	625	-
*C. limon*	“Siciliano” lemon	fruit peels	53% R-Limonene, 13% β-pinene, 4% citral	[23]	156	-
*C. limonia*	“Cravo” lime	fruit peels	65% R-Limonene, 9% β-pinene	[23]	625	-
*Croton cajucara*	White “sacaca”	leaves	28% Linalool	[24]	1250	-
*C. cajucara*	Red “sacaca”	leaves	18% Linalool, 25% 7-hydroxy-calamenene	[24]	2500	-
*Cymbopogon citratus*	Lemongrass	leaves	75% Citral	[22]	78	156
*Lippia alba* (LA10) *	Lemon balm	leaves	77% Citral	[25]	156	312
*L. alba* (LA13)	Lemon balm	leaves	45% Limonene, 40% Carvone	[25]	1250	-
*L. alba* (LA22)	Lemon balm	leaves	84% Linalool	[25]	156	156
*L. alba* (LA29)	Lemon balm	leaves	68% Citral	[25]	312	-
*L. alba* (LA44)	Lemon balm	leaves	82% Citral	[25]	78	78
*L. alba* (LA57)	Lemon balm	leaves	19% Limonene, 77% Carvone	[25]	625	-
*Lippia sidoides* (LSID001)	Pepper rosmarin	leaves	83% Thymol	[26]	625	-
*L. sidoides* (LSID003)	Pepper rosmarin	leaves	80% Thymol	[26]	625	-
*L. sidoides* (LSID004)	Pepper rosmarin	leaves	80% Thymol	[26]	1250	-
*L. sidoides* (LSID005)	Pepper rosmarin	leaves	76% Thymol	[26]	2500	-
*L. sidoides* (LSID006)	Pepper rosmarin	leaves	81% Thymol	[26]	625	-
*L. sidoides* (LSID104)	Pepper rosmarin	leaves	8% Thymol, 55% Carvacrol	[26]	2500	-
*L. sidoides* (LSID301)	Pepper rosmarin	leaves	76% Thymol	[26]	2500	-

- Not tested; * codes correspond to the accessions from the Active Germplasm Bank of the Federal University of Sergipe, Brazil.

**Table 3:** Determination of the minimum inhibitory concentration (MIC) of commercially-available major components of EO against *Desulfovibrio alaskensis* NCIMB 13491 and against SRB growth in produced water samples.

Major Components	Purity, Sigma Aldrich Reference Number	MIC Against *D. alaskensis* (µg/mL)	MIC in Produced Water (µg/mL)
Citral	95%, C83007	78	78
Linalool	97%, L2602	78	625
Geraniol	98%, 163333	78	312
Nerol	~98%, N7761	625	-
Eugenol	99%, E51791	78	312
R-Limonene	97%, 183164	2500	-
S-Limonene	96%, 218367	2500	-

- Not tested.

## Supplementary Materials

Supplementary Materials are available.
