# Supplementary material for: Growth Inhibition of Sulfate-Reducing Bacteria in Produced Water from the Petroleum Industry Using Essential Oils
Source: Molecules. 2017 Apr 19;22(4):648. doi: 10.3390/molecules22040648 (PMC6153933; doi:10.3390/molecules22040648)

Figure S1. Dendrogram based on DGGE profiles (16S rRNA coding gene fragments) of the four produced water samples received from Petrobras Ilha Grande Bay Oil Terminal (TEBIG) using the Pearson similarity coefficient and the UPGMA clustering method.

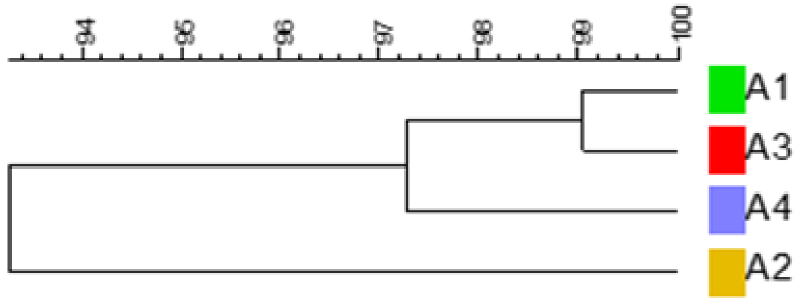

Supplement: Supplementary file 1 [file molecules-22-00648-s001.pdf]
